# Long-term effectiveness and safety of benralizumab in EGPA: a 3-year single-center experience

**DOI:** 10.1080/07853890.2025.2581812

**Published:** 2025-11-03

**Authors:** Federica Davanzo, Luca Iorio, Marta Codirenzi, Eleonora Fiorin, Gabriella Guarnieri, Alessia Achille, Fulvia Chieco Bianchi, Maria Rita Marchi, Andrea Vianello, Andrea Doria, Roberto Padoan

**Affiliations:** a Rheumatology Unit, Department of Medicine DIMED, University of Padova, Padua, Italy; b Respiratory Pathophysiology Division, Department of Cardiac-Thoracic-Vascular Sciences, University of Padua, Padua, Italy; c Respiratory Unit, Cittadella Hospital, Padua, Italy

**Keywords:** Asthma, Benralizumab, Eosinophilic granulomatosis with polyangiitis, Vasculitis

## Abstract

**Objectives:**

Benralizumab emerged as a promising treatment option for eosinophilic granulomatosis with polyangiitis (EGPA). This study assessed the long-term effectiveness and safety of benralizumab in patients with severe asthma and relapsing-refractory EGPA.

**Methods:**

This retrospective, single-center study evaluated patients treated with benralizumab (30 mg/8 weeks), followed for up to 36 months. Primary outcome included disease remission (defined as Birmingham Vasculitis Activity Score version 3 = 0 and prednisone dose ≤4 mg/day). Secondary endpoints were corticosteroid tapering, lung function, relapses, treatment failure and drug retention rates.

**Results:**

The study included 33 EGPA patients (17 [51.5%] male; median age at benralizumab initiation 56 years [IQR: 47–62]). Before starting benralizumab, most patients were on corticosteroids (90.9%), prior treatments included mepolizumab (24.2%). Benralizumab showed effectiveness, with clinical remission rates increasing from 39.4% (95% CI: 22.9–57.9) at 3 months to 65.0% (95% CI: 40.8–84.6) at 36 months (*p* < 0.001). Corticosteroid use declined from 90.9% to 15.4%, eosinophil counts dropped from 850 (515–1367) to 0 (0–0) cells/µL, and BVASv3 decreased from 2 (2–5) to 0 (0–0), showing significant improvements (*p* = 0.002 and *p* < 0.001, respectively). Proportion of patients experiencing asthma exacerbations reduced, alongside improved lung function. Retention rates were 81.8% at 1 year, 72.6% at 2 years, and 62.4% at 3 years, with secondary failure due to uncontrolled sinonasal symptoms. Mild adverse events were observed in 21.2% of patients.

**Conclusions:**

These findings support the long-term effectiveness and safety of benralizumab for EGPA, highlighting its role in inducing clinical remission, reducing corticosteroid dependence, and controlling disease activity.

## Introduction

Eosinophilic granulomatosis with polyangiitis (EGPA) is an antineutrophil cytoplasmic antibody (ANCA)-associated vasculitis characterized by chronic asthma, ear–nose–throat (ENT) symptoms, necrotizing vasculitis, extravascular granulomas, and elevated eosinophil levels in blood and tissues. The presence of ANCA, mostly directed against myeloperoxidase (MPO), has been reported in 30%–40% of the cases [1–3]. The pathogenesis involves a type 2 helper T-cell-driven immune response, with elevated interleukin 5 (IL-5) promoting eosinophil maturation, activation and survival [4] resulting in eosinophilic tissue infiltration and damage [5]. In parallel, a small vessel vasculitic process, mediated by ANCA and activated neutrophils, drives endothelial injury and, in some cases, granuloma formation [6].

A significant proportion of EGPA patients have a history of chronic rhinosinusitis with nasal polyps and adult-onset severe eosinophilic asthma (SEA) [7], conditions that often precede vasculitis and granuloma formation [8].

Oral corticosteroids (OCS) are the cornerstone of EGPA treatment, often combined with immunosuppressants for severe or refractory cases [9]. While effective for most patients, long-term OCS use is associated with persistent symptoms, frequent relapses, and significant side effects [9,10]. Immunosuppressants show limited efficacy in controlling eosinophilic disease and reducing OCS dependence [11].

Targeted therapies have shifted the treatment paradigm toward eosinophil-directed strategies [11–17]. Mepolizumab, a humanized monoclonal antibody targeting circulating IL-5, administered at 300 mg every 4 weeks, has proven effective in treating patients with relapsing-refractory EGPA while acting as a corticosteroid-sparing agent [18–20]. Benralizumab, a humanized monoclonal antibody targeting the IL-5 receptor alpha (IL-5Rα), effectively depletes eosinophils, eosinophil progenitors, and basophils *via* antibody-dependent cell-mediated cytotoxicity (ADCC) driven by natural killer cells [8].

Benralizumab, approved for SEA at a dose of 30 mg administered every 4 weeks for the first three doses, followed by every 8 weeks, has demonstrated efficacy in reducing exacerbations, OCS use, and blood eosinophil levels while improving lung function, asthma control, and clinical symptoms [21–23], with long-term data supporting a durable treatment response [24].

Benralizumab, at 30 mg every 8 weeks, was also effective in treating SEA associated with long-standing EGPA [25,26], with retrospective multicenter studies reporting improvements in respiratory and ENT symptoms, as well as reductions in OCS and immunosuppressant use [27–29]. A phase III randomized controlled trial confirmed that benralizumab (30 mg every 4 weeks) was non-inferior to mepolizumab (300 mg every 4 weeks) for remission maintenance in relapsing or refractory EGPA. This trial also showed benralizumab’s potential for greater corticosteroid-sparing effects, reporting higher rates of OCS withdrawal (41% vs. 26%) and dose reduction (86% vs. 74%) [30].

In 2020, we published one of the first case series demonstrating the efficacy of benralizumab in five patients with long-standing relapsing EGPA [25]. The present study provides additional evidence on the effectiveness and safety of benralizumab in a larger cohort of EGPA patients supported by long-term data.

## Methods

### Study design and participants

This was a retrospective, single-center, observational study conducted in patients with an established clinical diagnosis of EGPA treated in a multidisciplinary setting at Padua University Hospital.

The study enrolled adult patients diagnosed with severe asthma and relapsing-refractory EGPA who began treatment with benralizumab between July 2019 and January 2023. Patients received benralizumab at a dose of 30 mg every 8 weeks, following an initial phase of three doses administered every 4 weeks, as per the dosage approved for SEA. The treatment was used at any line of therapy. The decision to administer benralizumab was based on clinical judgment, independent of patient participation in the study, and according to the Italian eligibility criteria for benralizumab prescription in SEA [31], as follows: confirmed diagnosis of SEA with a baseline peripheral blood eosinophil count ≥300 cells/µL in the absence of systemic steroid treatment, and a minimum of two exacerbations in the prior year despite maximal inhaled therapy (GINA step 4–5 treatment) [32] treated with systemic steroid or requiring hospitalization and/or maintenance OCS therapy.

The OCS tapering protocol was guided by clinical judgment and tailored to the specific characteristics of each patient.

Relapsing-refractory EGPA was defined according to the European Alliance of Associations for Rheumatology (EULAR) 2022 recommendations [9]; severe asthma [33] and difficult-to-treat asthma [32] were defined according to the European Respiratory Society/American Thoracic Society (ERS/ATS) guidelines.

Exclusion criteria included patients with vasculitis manifestations who were not receiving concomitant immunosuppressive therapy, patients concurrently on other biologics, and those with comorbidities requiring chemotherapy or radiotherapy within the previous 12 months.

The study was approved by the Ethics Committee of Padua University Hospital (EC protocol n. 0060949, 11 November 2024), and all the patients provided informed consent for participation in the study and the use of their data retrospectively, from the date of consent back to the onset of their disease.

### Study objectives and measures

The primary objective of the study was the achievement of clinical remission during follow-up, as defined in the MIRRA and MANDARA trials [18,30], characterized by the absence of disease activity (Birmingham Vasculitis Activity Score version 3 (BVASv3) [34] of 0) and a daily oral prednisone dose of ≤4 mg/day. Disease relapse, assessed only in patients who had previously achieved remission, was defined according to the MIRRA and MANDARA trials as meeting at least one of the following criteria: active vasculitis (BVAS >0), initiation of a new immunosuppressant, worsening asthma or ENT symptoms requiring an increase in OCS dose >4 mg/day, or hospitalization [18,30]. Treatment failure was classified as primary when patients failed to achieve a complete response, and as secondary when there was a worsening of asthma and/or ENT manifestations or a vasculitis flare, requiring an escalation in immunosuppressive therapy following an initial response to benralizumab.

Secondary objectives included reduction of OCS use, respiratory function, discontinuation rates, causes of treatment failure, as well as overall safety.

Demographic, clinical, and laboratory data were collected retrospectively at baseline (start of the benralizumab treatment) and 3, 6, 12, 24 and 36 months. Baseline data included patient demographics and disease characteristics at diagnosis and the initiation of benralizumab therapy. Throughout the follow-up period, all patients underwent multidisciplinary assessments, which included pneumological, allergological, rheumatological, and rhinological evaluations. During each respiratory evaluation, a pulmonary function test was performed following international guidelines with standardized protocols [35], and data collected included pre-bronchodilator forced expiratory volume in 1 s (FEV_1_, measured in liters/second and as a percentage), forced vital capacity (FVC, in liters and as a percentage), and forced expiratory flow at 25%–75% (FEF 25–75). Fractional exhaled nitric oxide (FeNO, measured in ppb) was also recorded when available. The severity of asthma-related symptoms was evaluated using the Asthma Control Test (ACT) score [36], whereas nasal symptoms were assessed with the Sinonasal Outcome Test-22 (SNOT-22) score [37], collected during rhinological evaluation.

Respiratory outcomes included the evaluation of asthma exacerbations, defined according to the European Respiratory Society and European Academy of Allergy and Clinical Immunology statement [38]. Exacerbations were identified by one or more of the following: an asthma attack requiring an increase in OCS dose, an asthma-related emergency department admission, and/or the use of acute OCS, antibiotics, or short-acting beta-agonists [38]. Safety data were collected at every visit, with serious events defined as those resulting in death, requiring hospitalization, or causing significant disability [39].

### Statistical analysis

Qualitative variables are presented as absolute numbers and percentages, while continuous variables are reported as median and interquartile ranges (IQRs). Analyses were restricted to patients with data available at each specific time point. For each variable and time point, the total number of available observations is reported when missing data were present.

The normality of data distribution was assessed using the Shapiro-Wilk test. As the data did not meet the assumptions of normality, non-parametric tests were applied.

Comparative analyses were performed to evaluate the effectiveness of benralizumab across different time-points relative to baseline, as well as between various patient groups.

Remission rates were calculated as the proportion of patients meeting the criteria, with each time point analyzed independently of prior response status. Response rates were expressed as relative frequencies (%), and an exact binomial test with Clopper–Pearson 95% CIs was performed. At each follow-up time point (months 3, 6, 12, 24 and 36), the proportion of patients in remission was compared to baseline using *χ*2 test. According to the Bonferroni correction for multiple comparisons across the five time points, *p*-values ≤0.010 were considered statistically significant.

For the secondary analyses, continuous variables were compared across the five follow-up time points using the nonparametric Friedman test for repeated measures from months 0 to 36, followed by Dunn’s multiple comparisons test to compare each follow-up time point (months 3, 6, 12, 24 and 36) with baseline (month 0), with an additional Bonferroni correction applied for multiple testing. Categorical variables were analyzed using the *χ*^2^ test or Fisher’s exact test, as appropriate, with *p*-values ≤0.002 considered statistically significant based on the Bonferroni correction for multiple comparisons.

Kaplan–Meier survival analysis was used to assess the cumulative retention rate of benralizumab, with drug discontinuation due to inefficacy/adverse event defined as the endpoint.

All the analyses were performed using the free software Jamovi (version 2.4.11) and SPSS Version 26.0 software package (SPSS Inc., Chicago, IL, USA, 2001).

## Results

### Demographic and clinical characteristics

Patient demographic and clinical characteristics at EGPA diagnosis and at the beginning of benralizumab treatment are listed in Table 1.

**Table 1.** Patient demographic and clinical characteristics at EGPA diagnosis and initiation of benralizumab treatment.

EGPA patients (*n* = 33)	At EGPA diagnosis	At initiation of benralizumab
Male	17 (51.5%)	–
Age (years)	52 (41–48)	56 (47–62)
Anti-MPO ANCA	11 (33.3%)	3 (9.1%)
Eosinophil count (cells/µL)	1980 (1360–4440)	850 (515–1367)
BVASv3	25 (23–26)	2 (2–5)
FFS ≥1	2 (6.1%)	0 (0%)
Disease duration (months)	–	31 (16–94)
Risk factors		
Carcinogen exposure	5 (15.2%)	–
Atopy	16 (48.5%)	–
Alcohol	15 (45.5%)	–
Previous smoking habit	8 (24.2%)	–
Clinical involvement		
General symptoms	15 (45.5%)	6 (18.2%)
Pulmonary infiltrates	19 (57.6%)	2 (6.1%)
Asthma	33 (100%)	29 (87.9%)
ENT	33 (100%)	22 (66.7%)
Cutaneous	7 (21.2%)	2 (6.1%)
Cardiac	1 (3%)	0 (0%)
Gastrointestinal	0 (0%)	0 (0%)
Renal	1 (3%)	0 (0%)
Peripheral neuropathy	11 (33.3%)	5 (15.2%)
Central nervous system	1 (3%)	0 (0%)
Treatments from EGPA diagnosis until the initiation of benralizumab treatment		
Oral corticosteroids	30 (90.9%)	
OCS daily dose (mg/day)	7.5 (5–12.5)	
Patients on OCS ≥7.5 mg/day	18 (54.5%)	
LABA and/or ICS	33 (100%)	
Beclomethasone	16 (48.5%)	
Fluticasone	7 (21.2%)	
Budesonide	10 (30.3%)	
Low dose	1 (3.0 %)	
Medium dose	7 (21.2 %)	
High dose	25 (75.7%)	
LAMA	25 (75.7%)	
Previous anti-leukotriene agents	3 (9.1%)	
Local nasal medications	30 (90.9%)	
Intranasal corticosteroids	24 (72.7%)	
Antihistamines	26 (78.8%)	
Saline irrigations	30 (90.9%)	
Other biologic agents	0 (0%)	
Previous ESS	22 (66.7 %)	
Mepolizumab 300 mg/4 weeks	3 (9.1%)	
Mepolizumab 100 mg/4 weeks	5 (15.2%)	
Cyclophosphamide	2 (6.1%)	
Azathioprine	11 (33.3%)	
Mycophenolate mofetil	2 (6.1%)	
Methotrexate	17 (51.5%)	
Rituximab	2 (6.1%)	

Note: Data are expressed as *n* (%) and median (IQR). Percentage are calculated based on available data.

Abbreviations: ANCA: antineutrophil cytoplasmic antibodies; BVASv3: Birmingham Vasculitis Activity Score version 3; EGPA: eosinophilic granulomatosis with polyangiitis; ENT: ear, nose, and throat; ESS: Endoscopic Sinus Surgery; FFS: Five-Factor Score; ICS: inhaled corticosteroid; LABA: long-acting beta agonist; LAMA: long-acting muscarinic antagonist; MPO: myeloperoxidase; OCS: oral corticosteroids.

#### Patient characteristics at EGPA diagnosis

We included 33 patients with severe asthma and relapsing-refractory EGPA who began treatment with benralizumab between July 2019 and January 2023. Patients had a median age of 52 (41–48) years and 51.5% were male. At the time of diagnosis, 11 (33.3%) patients were positive for ANCA with anti-MPO specificity, and the median eosinophil count was 1980 (1360–4440) cells/µL. BVASv3 at diagnosis was 24.5 (23.1–25.9), and two patients (6.1%) had a Five-Factor Score of at least one. With regards to the risk factors, five (15.2%) patients had a history of exposure to carcinogenic substances, 16 (48.5%) were atopic, 15 (45.5%) had a history of alcohol consumption, and eight (24.2%) were previous smokers. The median duration of asthma before EGPA diagnosis was 8 (2–22) years (Table 1).

#### Patient characteristics at the beginning of benralizumab treatment

At the beginning of benralizumab treatment, the median disease duration was 31 (16–94) months, with the most common manifestations being severe asthma (87.9%) and ENT symptoms (66.7%). Other manifestations included general symptoms (18.2%), peripheral neuropathy (15.2%), pulmonary infiltrates (6.1%), and cutaneous manifestations (6.1%).

At the initiation of benralizumab (Table 1), nearly all patients were receiving OCS (90.9%), with 54.5% on doses of 7.5 mg/day or higher. Benralizumab was started on top of conventional immunosuppressants in six (7.8%) cases (*n* = 1 on mycophenolate mofetil, *n* = 5 on methotrexate). The most commonly used therapies prior to benralizumab were methotrexate (51.5%) and azathioprine (33.3%). Notably, eight patients (24.2%) had previously been treated with mepolizumab, either 300 mg (*n* = 3) or 100 mg (*n* = 5). Among those switching from mepolizumab 100 mg, the main reasons were uncontrolled asthma (*n* = 4) and ENT-related symptoms (*n* = 4). All three patients who switched from mepolizumab 300 mg did so because of persistent asthma symptoms.

All patients were receiving inhaled corticosteroids (ICS) and a long-acting β2-adrenoceptor agonist (LABA) at the time of benralizumab initiation. Additionally, 75.7% of patients were on long-acting muscarinic antagonist (LAMA) therapy, while none were actively taking anti-leukotriene agents. Only 9.1% of patients had a history of anti-leukotriene treatment. Furthermore, 90.9% of patients were also using local nasal medications, including combination of intranasal corticosteroids, antihistamines or saline irrigations. None of the patients were receiving other biologic therapies for nasal polyposis. Finally, 66.7% of patients had undergone at least one endoscopic sinus surgery (ESS) prior to benralizumab initiation, while none required surgical intervention during the follow-up period.

At the beginning of benralizumab treatment, all patients except one had a BVASv3 ≥ 0. One patient started benralizumab with a BVASv3 of 0, using it as sequential maintenance therapy within 6 months after achieving rituximab-induced remission, which was required due to the severity of their initial disease presentation.

### Effectiveness of benralizumab

#### Clinical remission

**Figure 1.**
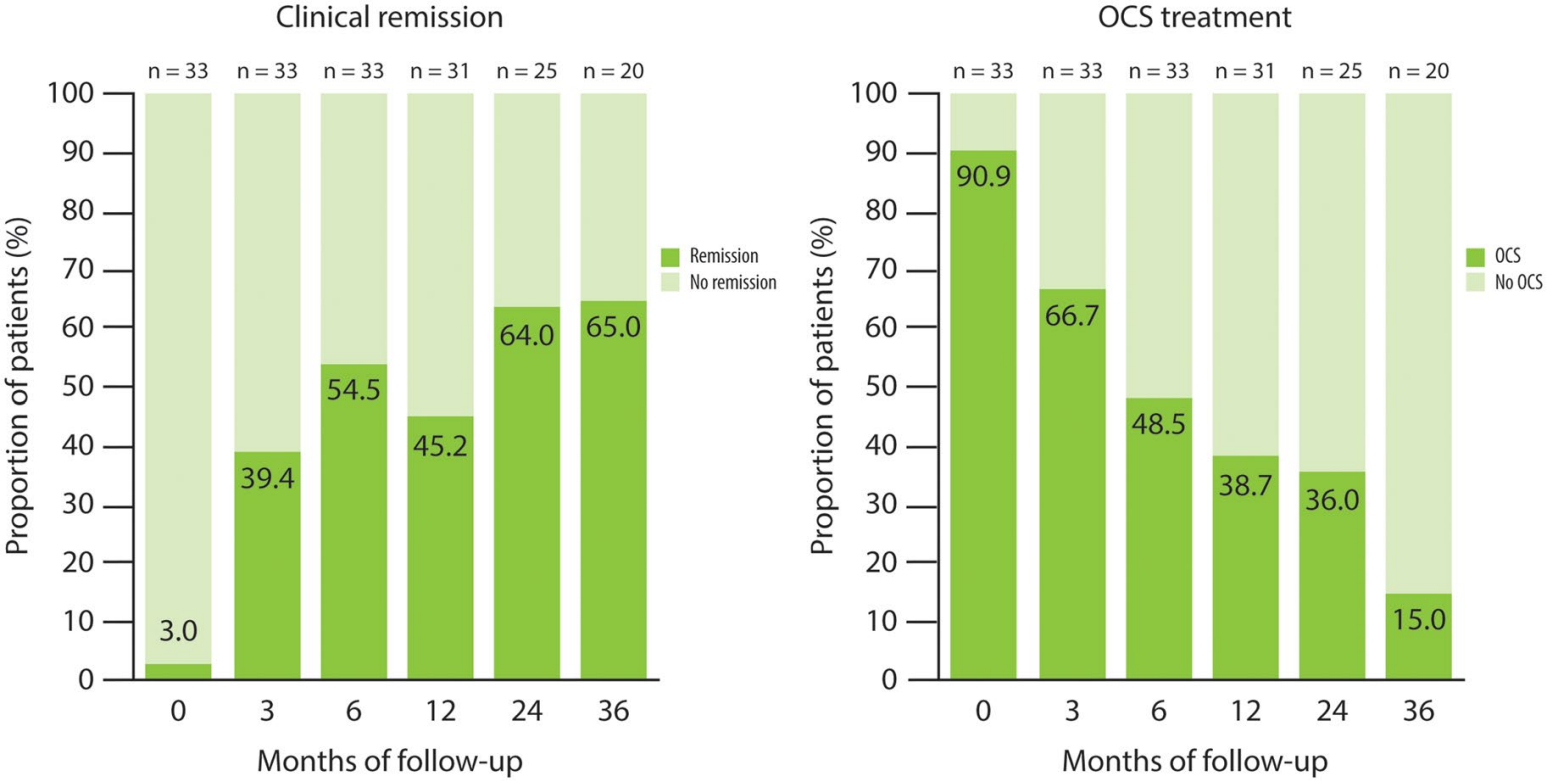
**Remission rate and ongoing oral corticosteroids use following benralizumab treatment.** Clinical remission was defined as Birmingham Vasculitis Activity Score version 3 of 0 and oral prednisone dose ≤4 mg/day. Clinical remission was achieved by a progressively increasing proportion of patients, starting 3 months after treatment initiation and peaking by 36 months (*p* < 0.001 compared with baseline across all timepoints). A significant progressive reduction in the proportion of patients using OCS was observed throughout the entire follow-up period (*p* < 0.001 compared with baseline across all timepoints).

Clinical remission was achieved by a progressively increasing proportion of patients, starting 3 months after treatment initiation and peaking by 36 months. Specifically, 13/33 patients (39.4%, 95% CI: 22.9–57.9) achieved remission after 3 months, 18/33 (54.5%, 95% CI: 36.4–71.9) at 6 months, 14/31 (45.2%, 95% CI: 27.3–64.0) at 12 months, 16/25 (64.0%, 95% CI: 42.5–82.0) at 24 months and 13/20 (65.0%, 95% CI: 40.8–84.6) at 36 months. These results showed significant improvement compared with baseline across all time points (*p* < 0.001) (Figure 1A and Table 2). Longitudinal transitions in clinical remission status among EGPA patients treated with benralizumab are illustrated in a Sankey plot, which depicts patient flow across baseline and follow-up visits at 3, 6, 12, 24 and 36 months (Supplementary Figure 1).

**Table 2.** Changes in OCS use and concomitant therapies during follow-up.

Outcome	Baseline (*n* = 33)	3 months (*n* = 33)	*p-*value	6 months (*n* = 33)	*p-*value	12 months (*n* = 31)	*p-*value	24 months (*n* = 25)	*p-*value	36 months (*n* = 20)	*p-*value
OCS (mg/day)	7.5 (5–12.5)	5 (0–5)	0.607	0 (0–5)	**0.011**	0 (0–5)	**0.005**	0 (0–5)	**0.006**	0 (0–0)	**<0.001**
Patients on OCS	30/33 (90.9%)	22/33 (66.7%)	**<0.001**	16/33 (48.5%)	**<0.001**	12/31 (38.7%)	**<0.001**	9/25 (36.0%)	**<0.001**	3/20 (15.0%)	**<0.001**
ICS/LABA	33/33 (100.0%)	33/33 (100.0%)	1.000	33/33 (100.0%)	1.000	31/31 (100.0%)	1.000	25/25 (100.0%)	1.000	19/20 (95.0%)	0.195
LAMA	27/33 (75.5%)	21/33 (63.6%)	0.097	22/33 (66.7%)	0.159	21/31 (67.7%)	0.194	15/25 (60.0%)	0.065	11/20 (55.0%)	0.036
Local nasal medication use	30/33 (90.9%)	30/33 (90.9%)	1.000	30/33 (90.9%)	1.000	28/31 (90.3%)	0.938	21/25 (87.5%)	0.424	13/20 (65.0%)	0.019

Note: Data are expressed as n (%) and median (IQR). Each follow-up time point (months 3, 6, 12, 24 and 36) is compared with the baseline. Categorical variables were compared using the χ^2^ test with Bonferroni correction for multiple comparisons; continuous variables were analyzed using the Friedman test, followed by Dunn’s multiple comparisons test with an additional Bonferroni correction applied for multiple testing. Statistically significant p-value according to post hoc tests are reported in bold.

Abbreviations: ICS: inhaled corticosteroid; IS: immunosuppressants; LABA: long-acting beta agonist; LAMA: long-acting muscarinic antagonist; OCS: oral corticosteroids.

#### Corticosteroid weaning

A significant progressive reduction in the proportion of patients using OCS was observed throughout the entire follow-up period (*p* < 0.001). At baseline, 90.9% of patients were on OCS, decreasing significantly to 66.7% at 3 months (*p* < 0.001), and continuing to decline to 48.5% at 6 months (*p* < 0.001), 38.7% at 12 months (*p* < 0.001), 36.0% at 24 months (*p* < 0.001), and 15.0% at 36 months (*p* < 0.001) (Figure 1B and Table 2).

The median daily OCS dose also decreased significantly over the follow-up period (*p* < 0.001), from 7.5 (5–12.5) mg/day at baseline to 5 (0–5) mg/day at 3 months (*p* = 0.607), and further to 0 (0–5) mg/day at 6, 12 and 24 months (*p* = 0.011, *p* = 0.005, *p* = 0.006, respectively). By 36 months, the median dose had dropped to 0 (0–0) mg/day, with a statistically significant difference from baseline (*p* < 0.001). Furthermore, 83.3% of patients discontinued immunosuppressants during follow-up. The use of local nasal medications and LAMA remained relatively consistent throughout the follow-up period, with no significant changes observed (local nasal medications: 90.9% at baseline vs. 65.0% at 36 months; LAMA: 67.7% at baseline vs. 55.0% at 36 months). All patients remained on ICS/LABA therapy throughout follow-up, and 4 out 33 patients (12.1%) were permitted to reduce the ICS dosage while continuing the combination treatment (Table 2).

#### Disease activity, eosinophil count, pulmonary function test and damage accrual

**Figure 2.**
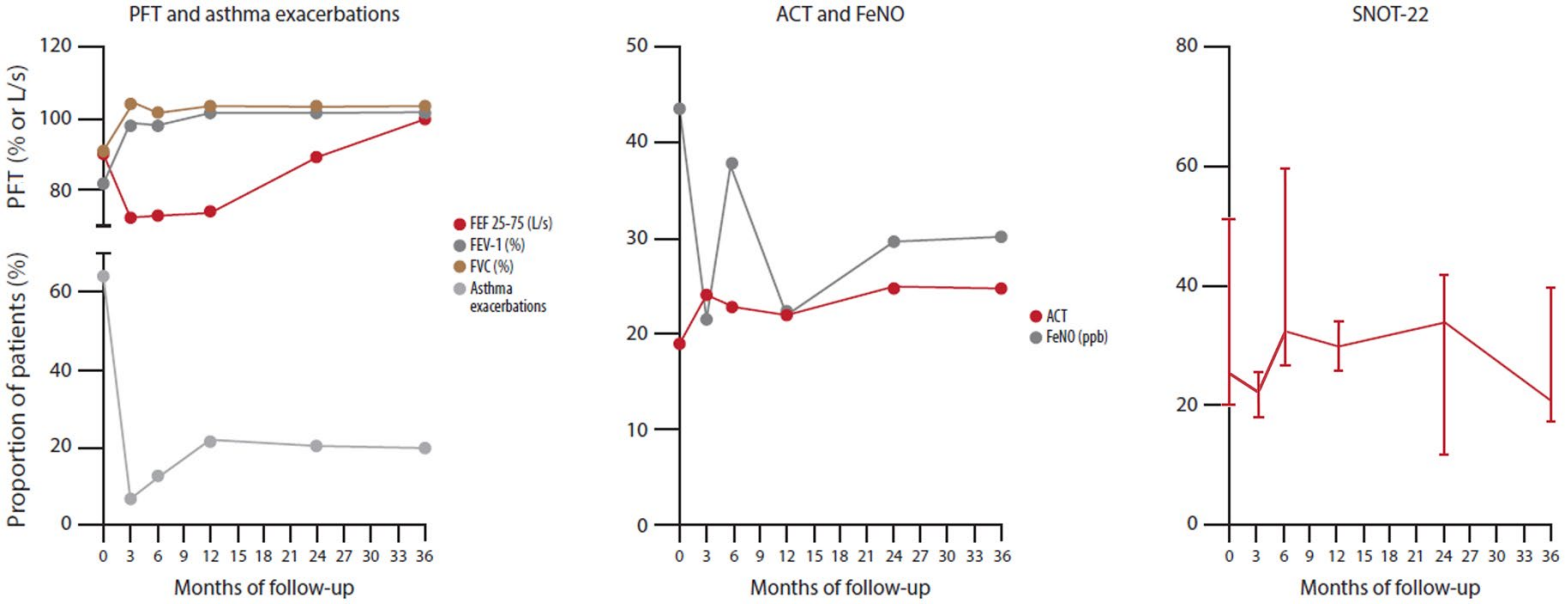
**Pulmonary function tests (PFT), asthma exacerbations, and patient-reported outcomes following benralizumab treatment.** Pulmonary function tests, including FEV1%, FVC%, FEF 25–75%, and ACT scores showed significant improvement over the follow-up period (*p* < 0.001, *p* = 0.005, *p* < 0.001 and *p* = 0.010 respectively). FeNO levels and SNOT-22 scores did not show significant changes. The incidence of asthma exacerbations decreased significantly (*p* = 0.002) during follow-up.

The results for disease activity, eosinophil counts, pulmonary function tests and damage accrual are summarized in Table 3 and in Figure 2. A significant reduction in both circulating eosinophil counts and BVASv3 score was observed throughout the entire follow-up period (*p* < 0.001 for both). As early as 3 moths, the eosinophil count dropped from 850 (515–1367) cells/µL to 0 (0–0) cells/µL, *p* = 0.002. Similarly, the BVASv3 score decreased significantly from 2 (2–5) to 0 (0–0), *p* < 0.001. When assessing VDI variation over the entire follow-up using the Friedman test, a significant difference was observed (*p* = 0.017), however no significant differences were found between individual time points and baseline.

**Table 3.** Longitudinal changes in eosinophil count, disease activity, damage accrual, pulmonary function, and patient-reported outcomes following benralizumab treatment.

Outcome	Baseline (*n* = 33)	3 months (*n* = 33)	*p-*value	6 months (*n* = 33)	*p-*value	12 months (*n* = 31)	*p-*value	24 months (*n* = 25)	*p-*value	36 months (*n* = 20)	*p-*value
Eosinophil count (cells/µL)	850 (515–1367)	0 (0–0)	**0.002**	0 (0–0)	**0.002**	0 (0–0)	**0.008**	0 (0–5)	**0.008**	0 (0–0)	**0.008**
Patients with asthma exacerbations	21/33 (63.6%)	2/33 (6.1%)	**<0.001**	4/33 (12.1%)	**<0.001**	7/31 (21.9%)	**0.001**	5/25 (20.0%)	**<0.001**	4/20 (20.0%)	**0.002**
BVASv3	2 (2–5)	0 (0–0)	**<0.001**	0 (0–0)	**<0.001**	0 (0–2)	**0.006**	0 (0–0)	**0.001**	0 (0–2)	**0.005**
VDI	3 (2–5)	4 (3–5)	1.000	4 (3–5)	1.000	4 (3–5)	1.000	4 (3–5)	1.000	4 (3–5)	1.000
FEV_1_ (%)	82 (65–95) *data on 31 patients	100 (83–107) *data on 28 patients	0.210	99 (85–106) *data on 29 patients	**0.020**	103 (85–109) *data on 29 patients	**0.001**	102 (85–110) *data on 24 patients	**<0.001**	103 (82–113) *data on 19 patients	**0.003**
FVC (%)	90 (81.5–102) *data on 31 patients	105 (88.5–113.5) *data on 28 patients	0.080	102 (97–114) *data on 29 patients	0.051	104 (91–113) *data on 29 patients	**0.027**	103 (91–109) *data on 24 patients	**0.005**	102 (89–114) *data on 19 patients	0.140
FEF 25–75 (L/s)	90 (81.5–102) *data on 28 patients	73 (60–91) *data on 25 patients	**0.025**	73.5 (52–91) *data on 24 patients	0.082	75 (59–118) *data on 19 patients	**0.012**	90 (56–110) *data on 15 patients	**0.012**	101 (58–118) *data on 18 patients	**<0.001**
FeNO (ppb)	44 (21.3–61.7) *data on 29 patients	22 (15.5–43) *data on 25 patients	**	37.7 (20–60.2) *data on 20 patients	**	21.9 (14.5–34.5) *data on 27 patients	**	29.8 (17.2–40.0) *data on 21 patients	**	30.3 (17.5–118) *data on 18 patients	**
ACT	19 (16–23) *data on 27 patients	24 (21–25) *data on 22 patients	1.000	23 (21–24) *data on 24 patients	0.547	22 (19–24) *data on 21 patients	0.107	25 (22–25) *data on 21 patients	**0.028**	25 (22–25) *data on 16 patients	0.214
SNOT-22	28 (20–49) *data on 16 patients	21 (18–26) *data on 4 patients	**	33 (27–61) *data on 7 patients	**	30 (26–34) *data on 2 patients	**	34 (13–42) *data on 8 patients	**	21 (18–40) *data on 12 patients	**

Note: Data are expressed as median (IQR). Each follow-up timepoint (months 3, 6, 12, 24, and 36) is compared with the baseline. Categorical variables were compared using the χ^2^ test with Bonferroni correction for multiple comparisons; continuous variables were analyzed using the Friedman test, followed by Dunn’s multiple comparisons test with an additional Bonferroni correction applied for multiple testing. Statistically significant p-value according to post hoc tests are reported in bold. ** Friedman test not significant; therefore, pairwise comparisons were not performed.

Abbreviations: ACT: Asthma Control Test; BVASv3: Birmingham Vasculitis Activity Score version 3; FEF: forced expiratory flow; FeNO: fractional exhaled nitric oxide; FEV_1_: forced expiratory volume; FVC: forced vital capacity; SNOT-22: Sino-Nasal Outcome Test-22; VDI: Vasculitis Damage Index.

At baseline, three patients were ANCA-positive, one of them became ANCA negative during follow-up, while no patient developed new ANCA positivity.

Pulmonary function tests, including FEV_1_%, FVC%, FEF 25%–75%, showed significant improvement over the follow-up period (*p* < 0.001, *p* = 0.005 and *p* < 0.001 respectively). ACT scores also improved (*p* = 0.010), increasing from a median of 19 (15.5–22.5) at baseline to 25 (22–25) at 36 months. Although FeNO levels did not change significantly during follow-up (*p* = 0.210), a downward trend was noted. The incidence of asthma exacerbations decreased significantly, from 63.6% of patients at baseline to 20.0% at 36 months (*p* = 0.002). In contrast, SNOT-22 scores did not show significant changes, decreasing slightly from 28 (20–49) at baseline to 21 (18–40) at 36 months. None of the patients required surgical procedures during the follow-up period.

#### Retention rates

The median treatment duration was 35 (20–36) months. The retention rate was 93.9% at 6 months, 81.8% at 12 months, 72.6% at 24 months, and 62.4% at 36 months.

During the follow-up period, 36.4% (*n* = 12) of patients discontinued benralizumab, half of them at 12 months (6/31), 2/33 at 6 months, 3/25 at 25 months and 1/20 at 36 months. The primary reasons for discontinuation are reported in Table 4. Primary failure was observed in 2 patients (16.7%), while secondary failure accounted for the majority of discontinuations, affecting 9 patients (75.0%). All patients with secondary failure (100%, *n* = 9) experienced uncontrolled ENT symptoms despite ongoing local nasal medications, and 16.7% (*n* = 2) also reported uncontrolled asthma. Vasculitis relapse (manifested as worsening peripheral neuropathy) was documented as the reason for discontinuation in 1 patient (8.3%). Pregnancy led to discontinuation in 1 patient (8.3%) due to safety considerations. Notably, no discontinuations were attributed to severe adverse events.

**Table 4.** Discontinuation rates and reasons at last follow-up.

Characteristics	*n* (%)
Patients discontinuing benralizumab	12/33 (36.4)
Reason for treatment discontinuation: Primary failure Secondary failure Uncontrolled ENT symptoms Uncontrolled asthma Vasculitis relapse Other (pregnancy) Severe adverse events Remission	2/12 (15.4%) 9/12 (75.0%) 9/9 (100%) 2/9 (22.2) 1/12 (8.3%) 1/12 (8.3%) 0 (0.0%) 0 (0.0%)
Shift to other biologics Mepolizumab 100 mg/4w Mepolizumab 300 mg/4w Dupilumab 300 mg/2w	10/12 (83.3) 0/10 (0) 5/10 (50.0) 5/10 (50.0)

Abbreviation: ENT: ear, nose, and throat.

Following discontinuation, most patients (83.3%, *n* = 10) transitioned to alternative biologic therapies, with mepolizumab 300 mg every 4 weeks (50.0%, *n* = 5) or dupilumab 300 mg every other week (50.0%, *n* = 5).

Patients with treatment failure had a significantly higher prevalence of skin involvement at diagnosis (41.7% vs. 9.5%, *p* = 0.030) than those without failure; other disease manifestations, clinical scores, and pulmonary function parameters were comparable between groups. No significant differences were observed in demographic characteristics, eosinophil counts, C-reactive protein levels, ANCA status, or ongoing treatments at the initiation of benralizumab (Supplementary Table 1).

### Safety of benralizumab

A total of seven patients (21.2%) experienced adverse events. During the follow-up period, nine mild adverse events were recorded, including five cases of headache, two cases of myalgia, one case of gastric pyrosis, and one patient reporting somnolence. Most events (7/9, 77.8%) occurred within the first 6 months of therapy, except for one episode of headache after 12 months and one episode of arthro-myalgia after 2 years of treatment. No definite causal association with benralizumab was identified, and no serious adverse events leading to hospitalization or treatment discontinuation were reported.

## Discussion

Benralizumab, administered at the approved dose for SEA (30 mg every 8 weeks), significantly improves clinical outcomes in patients with EGPA, supporting its use in a real-world setting for this population. Benralizumab proved effective in achieving sustained remission, reducing OCS and immunosuppressant use, lowering blood eosinophil counts, and improving pulmonary function tests and asthma control. These improvements were observed as early as 3 months after treatment initiation and sustained throughout the follow-up, including long-term outcomes. Furthermore, benralizumab was associated with high treatment retention rates and a favorable safety profile, with no new or unexpected adverse events reported. These findings align with those of other retrospective and prospective multicenter studies, as summarized in Table 5, as well as the results of the latest meta-analysis by Spataro and colleagues [25–30,40–42].

**Table 5.** Comparative summary of prospective and retrospective studies on benralizumab in EGPA.

Author, year	Cohort	Follow-up (m)	Previous me­po­li­zum­ab	ANCA-MPO positive	Remission (BVAS = 0 and OCS ≤ 4), %	Relapse, % (patients)	Adverse events, (patients or events)	Benralizumab discontinuation, %	OCS discontinuation, %
Guntur et al. 2020 [40]	Prospective monocentric study, *n* = 10	6	0 (0)	30	–	–	23 events SAE: 0	0	50
Padoan et al. 2020 [25]	Prospective monocentric study, *n* = 5	6	3 (60)	20	–	–	0	0	60
Nolasco et al. (2023) [29]	Retrospective multicentric study, *n* = 26	24	–	26.9	42.3 (at 12 m) 69.2 (at 24 m)	33.3 (at 24 m)	2 (7.7) SAE: 1 event	0	28.3
Cottu et al. 2023 [27]	Retrospective multicentric study, *n* = 68	23 (9–34)	31 (46)	29	49.3	18	3 (4) SAE: 6 events	20.9	38
Bettiol et al. 2023 [28]	Retrospective multicentric study, *n* = 121	6 (*n* = 101) 12 (*n* = 85)	32 (26)	28	46.4 (at 12 m)	5.0	19 (16) SAE: 3 events	13	–
Wechsler et al. 2024 [30],*	Benralizumab arm of MANDARA trial, *n* = 70	12	0 (0)	26	59	30	63 (90) SAE: 6%	1.4	41
Nanzer et al. 2024 [41]	Retrospective monocentric study, *n* = 70	12 (*n* = 70) 24 (*n* = 53)	17 (24.3)	27	67.1 (at 12 m) 67.9 (at 24 m)	38.5 (at 12 m) 56.6 (at 24 m)	- SAE: 0	0	67.9 (at 12 m) 68.3 (at 24 m)
Mummler et al. (2024) [42]	Retrospective monocentric study, *n* = 26	12	9 (34.6)	7.6	–	11.5	– SAE 0	15.3	37.5
Present study	Retrospective monocentric study, *n* = 33	12 (*n* = 31) 24 (*n* = 25) 36 (*n* = 20)	8 (24.2)	33.3	45.1 (at 12 m) 64.0 (at 24 m) 65.0 (at 36 m)	33.3	7 (21.2) SAE: 0	36.4	90

*Benralizumab 30 mg every 4 weeks. BVASv3: Birmingham Vasculitis Activity Score version 3; OCS: oral corticosteroid; SAE: severe adverse event.

The overall clinical remission rate observed in our cohort (65.0% after 36 months) is comparable to previously reported rates, which ranged from 46.4% at 12 months to 67.9% at 24 months (Table 5), as well as the 56.8% remission rate reported by Spataro et al. [26] Additionally, the significant OCS-sparing effect seen in this study is consistent with earlier evidence (Table 5). Of particular interest, the meta-analysis by Spataro et al. suggests that younger patients may derive greater OCS-sparing benefits from benralizumab, possibly due to a more robust immune response and fewer comorbidities [26].

Benralizumab demonstrated significant improvements in pulmonary function, including increases in FEV_1_%, FVC% and FEF 25%–75%. A reduction in FeNO levels was also observed; however, this was not consistently significant, likely because FeNO production is predominantly mediated by IL-13, which is not directly influenced by IL-5 receptor blockade [43].

The profound depletion of blood eosinophils observed throughout the study underscores the pivotal role of eosinophils in the clinical manifestations of EGPA. The remission outcomes suggest that eosinophil-driven inflammatory pathways are a key therapeutic target in patients with EGPA. However, the extent to which blood eosinophil depletion translates into clinical and therapeutic benefits remains incompletely understood. While eosinophils are recognized as crucial mediators in the pathogenesis of EGPA, it is still debated whether their depletion directly correlates with clinical improvement.

Despite favorable outcomes in asthma control, limited improvement was observed in nasal symptoms as measured by the ENT questionnaire. This limitation was accompanied by a notable rate of treatment discontinuation due to uncontrolled or relapsing ENT symptoms, which were often classified as secondary failure. Similar findings have been reported in previous studies, with ENT relapses documented in one out of 14 patients in the study by Cottu et al. [27] five out of six patients in the study by Bettiol et al. [28] and two out of three patients in the study by Mümmler et al. [42] One possible explanation for the suboptimal control of ENT symptoms may be the progressive tapering of corticosteroids, combined with the lack of optimized topical therapy for rhinitis or sinusitis. However, no specific differences were identified in the failure group. Another hypothesis is that benralizumab may have limited efficacy in targeting activated eosinophils within nasal polyps. This could be due to reduced expression of IL-5Rα on eosinophils in nasal polyp tissue, a process attributed to IL-5Rα cleavage mediated by matrix metalloproteinases [44]. Notably, in the MANDARA trial, mepolizumab and benralizumab exhibited the same effectiveness in managing sino-nasal symptoms, as assessed through the SNOT-22 questionnaire [30]. This raises the possibility that a higher dosage of benralizumab might be required to achieve a positive outcome on ENT symptoms in patients with EGPA, although this hypothesis would need to be tested in future trials.

These findings highlight the importance of a personalized treatment approach for patients with EGPA, emphasizing the need to select the most appropriate biologic agent based on patient and disease characteristics. Mümmler et al. [42] reported that using benralizumab during the induction therapy phase was associated with higher remission rates, as defined by the EULAR criteria [9], than its use during the maintenance phase. Notably, the study by Mümmler and colleagues had a lower proportion of ANCA-positive patients [42], similar to the MIRRA trial population [18]. Further research is warranted to explore predictive factors of non-responsiveness to benralizumab, including molecular and immunological biomarkers [45]. The deep depletion of eosinophils *via* ADCC could explain the efficacy of benralizumab in patients who failed mepolizumab therapy [46]. However, additional studies are needed to validate this hypothesis and clarify the potential therapeutic differences between these two anti-IL-5 biologics.

From a safety perspective, benralizumab was well tolerated, with only 21.2% of patients experiencing mild adverse events and no reports of adverse events leading to treatment discontinuation. This aligns with previously published data [26,47], including studies on patients with SEA [23,48–50]. The adverse event rate observed in our study is substantially lower than that reported in the MANDARA trial [30], in which 90% of patients treated with benralizumab experienced adverse events, including headache and arthralgia, with 6% experiencing serious adverse events. The favorable safety profile of benralizumab is particularly relevant for patients who achieve withdrawal from immunosuppressants and OCS, as prolonged use of these medications is linked to an increased risk of infection and other complications. Moreover, emerging evidence suggests that benralizumab may have a favorable safety profile during pregnancy [50]. Another area for future investigation is the potential clinical impact of different dosing regimens for benralizumab. For instance, the 30 mg every 4 weeks dosing regimen used in the MANDARA trial [30] may offer enhanced efficacy compared with the 30 mg every 8 weeks regimen used in this retrospective study. Further studies are needed to evaluate whether higher dosing frequency improves clinical outcomes in EGPA.

### Strengths and limitations

We acknowledge some limitations of this study, including its retrospective design, relatively small sample size, and the absence of a control group, which has also been noted in similar published cohorts. The study’s retrospective nature introduces the possibility of underreporting events, particularly due to missing data in the assessment of functional measures and patient-reported outcomes, and limits the ability to account for spontaneous fluctuations in disease activity associated with the natural course of EGPA. The small sample size also restricted our ability to perform subgroup analyses, such as comparisons based on ANCA status. Given the exploratory nature of the study and the limited sample size, p-values for some secondary outcomes should be interpreted with caution. The small sample size also limited our ability to perform subgroup analyses, such as comparisons based on ANCA status. Additionally, as a single-center study, it was not possible to account for variability in clinical management across different centers, and practice-based confounding factors may have influenced the results.

Despite these limitations, this study has several notable strengths. Most importantly, it is the first study in the available literature to provide long-term data on both the effectiveness and safety of benralizumab in EGPA, offering a considerably longer follow-up period compared to previously published studies. This extended follow-up enables a more robust evaluation of sustained outcomes and drug retention-rate. Furthermore, patients were managed by a multidisciplinary team comprising a pulmonologist, an otorhinolaryngologist, a rheumatologist, and an allergologist. This collaborative approach facilitated the comprehensive collection of functional data, including pulmonary function and ENT-related outcomes, which are often underreported in other studies. Additionally, the multidisciplinary approach helped address the limited sensitivity of BVASv3 in capturing chronic respiratory manifestations of EGPA, providing a more accurate reflection of patient-reported symptoms and disease activity [17].

## Conclusion

In conclusion, our retrospective, single-center, observational study highlights the long-term effectiveness and safety of benralizumab in the treatment of patients with EGPA. Administered at the dose approved for SEA, benralizumab induced clinical remission in the majority of patients, achieved profound depletion of blood eosinophils, significantly reduced disease activity, and improved pulmonary function. Additionally, benralizumab enabled a significant reduction in OCS use, while maintaining effective control of EGPA manifestations.

Future research should further investigate the potential clinical impact of higher doses of benralizumab, its efficacy in patients refractory to mepolizumab, and the specific disease characteristics that may predict greater clinical benefit. Notably, the limited response observed in patients with persistent ENT symptoms emphasizes the importance of tailoring biologic therapies to individual disease phenotypes. This limited response has been observed, to date, with the severe asthma dosage. This raises the possibility that a higher dosage of benralizumab might be required to achieve a positive outcome on ENT symptoms in patients with EGPA, although this hypothesis would need to be tested in future trials.

## Supplementary Material

Supplemental Material

## Data Availability

The data that support the findings of this study are available from the corresponding author upon reasonable request.
