# Supplementary material for: Long-term effectiveness and safety of benralizumab in EGPA: a 3-year single-center experience
Source: Ann Med. 2025 Nov 3;57(1):2581812. doi: 10.1080/07853890.2025.2581812 (PMC12584834; doi:10.1080/07853890.2025.2581812)
Supplement: Supplemental Material [file IANN_A_2581812_SM9820.docx]

**Supplementary Figure 1. Longitudinal transitions in clinical remission status among EGPA patients treated with benralizumab.** The Sankey plot illustrates changes in clinical status (clinical remission vs. active disease) over time in the EGPA patient cohort (n=33) receiving benralizumab. Time points include baseline and follow-up visits at 3, 6, 12, 24, and 36 months. The width of the flows represents the number of patients transitioning between states.


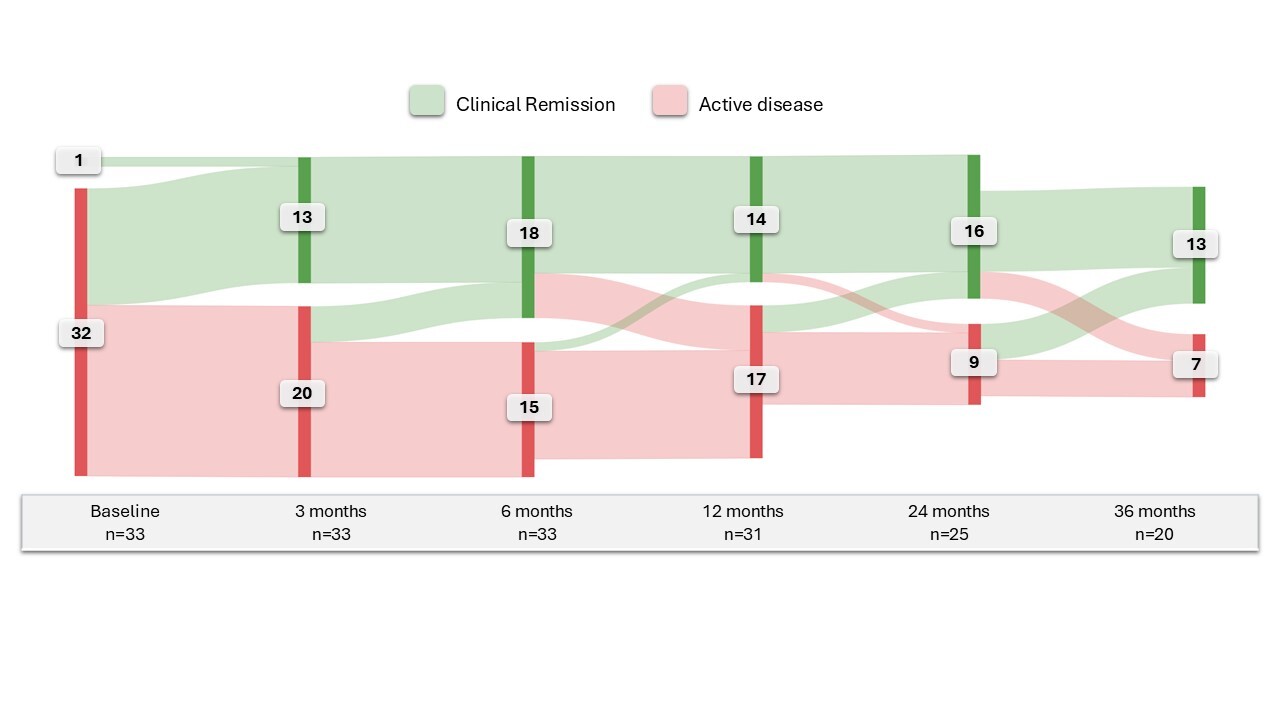


**Supplementary Table 1. Comparison of clinical and laboratory characteristics at diagnosis and benralizumab initiation between patients with and without treatment failure.**

|  | **Patients with treatment failure (n=12)** | **Patients without treatment failure (n=21)** | ***p-value*** |
| --- | --- | --- | --- |
| Age at diagnosis (years) | 48 (42–57) | 54 (41-60) | 0.476 |
| Age at benralizumab start (years) | 60 (48–63) | 55 (47–60) | 0.294 |
| Disease duration (months) | 91 (21–188) | 25 (16–42) | 0.160 |
| Eosinophil count at diagnosis (cells/µL) | 1590 (995–3820) | 2000 (1660–3630) | 0.261 |
| Eosinophil count at benralizumab start (cells/µL) | 860 (560–1290) | 689 (500–1390) | 0.648 |
| CRP at benralizumab start (mg/L) | 2 (0.6–3.2) | 2.9 (1.9–3.9) | 0.253 |
| Anti-MPO ANCA at diagnosis | 5 (41.7) | 6 (28.6) | 0.443 |
| Anti-MPO ANCA at benralizumab start | 1 (8.3) | 1 (4.8) | 0.679 |
| Comorbidities   - Airborne allergies - Alcohol use - Smoking history - BMI (kg/m^2^) | 6 (50.0)  6 (50.0)  4 (33.3)  1 (8.3)  24.5 (23.7–25) | 10 (47.6)  10 (47.6)  11 (52.4)  7 (33.3)  24.5 (22.8–28.4) | 0.710  0.710  0.359  0.133  0.896 |
| Disease manifestations at diagnosis   - Asthma - ENT involvement - Lung infiltrates - Peripheral neuropathy - Skin involvement - Systemic symptoms - Cardiac involvement - Central nervous system - Renal involvement | 12 (100)  12 (100)  7 (58.3)  5 (41.7)  5 (41.7)  6 (50.0)  0 (0)  1 (8.3)  1 (8.3) | 21 (100)  21 (100)  12 (57.1)  6 (28.6)  2 (9.5)  9 (42.9)  1 (4.8)  0 (0)  0 (0) | 1.000  1.000  0.947  0.443  **0.030**  0.692  0.443  0.179  0.179 |
| BVASv3 at benralizumab start | 2 (2–5) | 4 (2–4) | 0.829 |
| VDI at benralizumab start | 3 (2–5) | 3 (2–4) | 1.000 |
| ACT at benralizumab start | 19 (17–20) | 20 (15–23) | 0.877 |
| SNOT-22 at benralizumab start | 32 (26–52) | 24 (20–44) | 0.736 |
| Pulmonary function tests at benralizumab start   - FEV_1_ (%) - FVC (%) - FEF 25–75 (L/s) - FeNO (ppb) | 75.5 (48.2–93.8)  94.5 (87.8–102)  34.5 (18.2–65.8)  42.5 (25.1–44.6) | 82 (69–100)  90 (75–102)  54.5 (42.2–75.2)  44.7 (20.8–74.1) | 0.341  0.767  0.098  0.701 |
| Ongoing treatments at benralizumab start   - OCS - OCS dose (mg/day) - Methotrexate - Mycophenolate mofetil | 12 (100)  11 (5–12.5)  3 (25.0)  0 (0) | 18 (85.7)  5 (5–15)  2 (9.5)  1 (4.8) | 0.170  0.413  0.233  0.443 |

ACT: Asthma Control Test; BMI: body mass index; BVASv3: Birmingham Vasculitis Activity Score version 3; CNS: central nervous system; CRP: C-reactive protein; ENT: ear, nose, and throat; FeNO: fractional exhaled nitric oxide; FEF 25-75: forced expiratory flow at 25–75% of pulmonary volume; FEV_1_: forced expiratory volume in 1 second; FVC: forced vital capacity; MMF: mycophenolate mofetil; MPO: myeloperoxidase antibodies; MTX: methotrexate; OCS: Oral corticosteroids; SNOT-22: Sinonasal Outcome Test-22; VDI: Vasculitis Damage Index.
